# The Expression of Tubulin Cofactor A (TBCA) Is Regulated by a Noncoding Antisense *Tbca* RNA during Testis Maturation

**DOI:** 10.1371/journal.pone.0042536

**Published:** 2012-08-06

**Authors:** Sofia Nolasco, Javier Bellido, João Gonçalves, Alexandra Tavares, Juan Carlos Zabala, Helena Soares

**Affiliations:** 1 Departamento de Química e Bioquímica, Centro de Química e Bioquímica, Faculdade de Ciências, Universidade de Lisboa, Lisboa, Portugal; 2 Departamento de Biología Molecular, Facultad de Medicina, IFIMAV-Universidad de Cantabria, Santander, Spain; 3 Instituto Gulbenkian de Ciência, Oeiras, Portugal; 4 Escola Superior de Tecnologia da Saúde de Lisboa, Lisboa, Portugal; University of Maryland School of Medicine, United States of America

## Abstract

**Background:**

Recently, long noncoding RNAs have emerged as pivotal molecules for the regulation of coding genes' expression. These molecules might result from antisense transcription of functional genes originating natural antisense transcripts (NATs) or from transcriptional active pseudogenes. TBCA interacts with β-tubulin and is involved in the folding and dimerization of new tubulin heterodimers, the building blocks of microtubules.

**Methodology/Principal Findings:**

We found that the mouse genome contains two structurally distinct *Tbca* genes located in chromosomes 13 (*Tbca*13) and 16 (*Tbca*16). Interestingly, the two *Tbca* genes albeit ubiquitously expressed, present differential expression during mouse testis maturation. In fact, as testis maturation progresses *Tbca*13 mRNA levels increase progressively, while *Tbca*16 mRNA levels decrease. This suggests a regulatory mechanism between the two genes and prompted us to investigate the presence of the two proteins. However, using tandem mass spectrometry we were unable to identify the TBCA16 protein in testis extracts even in those corresponding to the maturation step with the highest levels of *Tbca*16 transcripts. These puzzling results led us to re-analyze the expression of *Tbca*16. We then detected that *Tbca*16 transcription produces sense and natural antisense transcripts. Strikingly, the specific depletion by RNAi of these transcripts leads to an increase of *Tbca*13 transcript levels in a mouse spermatocyte cell line.

**Conclusions/Significance:**

Our results demonstrate that *Tbca*13 mRNA levels are post-transcriptionally regulated by the sense and natural antisense *Tbca*16 mRNA levels. We propose that this regulatory mechanism operates during spermatogenesis, a process that involves microtubule rearrangements, the assembly of specific microtubule structures and requires critical TBCA levels.

## Introduction

Long noncoding RNAs have emerged as pivotal molecules for the regulation of coding genes' expression [1], [2], [3].

One of the classes of regulatory noncoding RNAs is that of natural antisense transcripts (NATs) that are defined as endogenous RNA molecules at least partially complementary to transcripts of established function [4]. Using genome wide approaches it became evident that antisense transcription is a widespread event throughout evolution [5], and that mammalian genomes encode a large number of NATs [6], [7], [8].

NATs can be classified in two major groups: *cis*-encoded, being produced from the same locus as their sense counterpart and *trans*-encoded being transcribed from different loci [9]. *Trans*-NATs also differ from *cis*-NATs by presenting inexact sequence complementarity with their sense counterpart transcript. In prokaryotes, NATs have been implicated in the control of plasmid replication, bacteriophage development and gene expression regulation [10], [11]. In eukaryotes, they are involved in transcription regulation [12]–[16], alternative splicing [17], [18], RNA stability and translation regulation, [15], [19]–[21], X-chromosome inactivation [22], [23], histone modification and DNA methylation in genomic imprinting [24]–[26]. The different biological functions carried out by NATs seem to involve a variety of distinct transcriptional and post-transcriptional regulatory mechanisms, such as recruitment/targeting of chromatin complexes, transcriptional interference, RNA masking, RNA editing, and RNA interference (RNAi) [5], [27], [28].

There are also growing evidences that long noncoding RNAs molecules might be produced by transcriptional active pseudogenes [29]. Traditionally, pseudogenes are considered as copies of protein-coding genes that have lost the ability to produce functional proteins [30] and it is well accepted that they can be created by diverse processes, like: (1) spontaneous mutations, preventing transcription of the gene or translation of the protein [31]; (2) duplication, in which pseudogenes are originated via tandem duplication or uneven crossing-over leading to the loss of promoters or enhancers or the appearance of crippling mutations such as frame shifts or premature stop codons [30] and (3) retrotransposition; the mRNA transcript is reverse-transcribed and integrated into the genome at a new location originating retrotransposed or processed pseudogenes [32], [33].

Recently, it was demonstrated that pseudogene transcripts can be processed into small interfering RNAs (siRNA) with the ability to repress gene expression in mouse oocytes [34], [35]. These siRNAs are derived either from pseudogenes with internal secondary structures, or from dsRNAs resulting from sense and antisense transcripts pairing. These mechanisms seem also to operate in plant genomes, for example in rice, in which a small number of pseudogenes are transcribed and processed into siRNAs, after pairing with the coding gene or a paralogous pseudogene transcript [36].

The regulatory role of NATS and transcribed pseudogenes seems to have a wide impact in testis where these RNAs are highly abundant [37]. The production of functional gametes in testis is a complex developmental and maturation process that requires, for example rearrangements of the microtubule cytoskeleton and the assembly of specialized microtubule structures [38]. Microtubules are polarized and dynamic polymers of α/β-tubulin heterodimers, that are involved in a great variety of cellular functions, e.g. the intracellular spatial organization, generation of cell polarity, intracellular transport, cell division, cell signalling and cell motility [39].

TBCA interacts with β-tubulin and together with other molecular chaperones and tubulin cofactors (TBCs, A–E) is involved in the maturation of new tubulin heterodimers [40]–[42]. Although not essential for tubulin heterodimer formation in *vitro* [42], TBCA knockdown by RNAi in human cell lines, leads to decreased amounts of α- and β-tubulin levels, subtle alterations in the microtubule cytoskeleton, G1 cell cycle arrest and cell death [43]. Previous studies focused in *Tbca* expression have shown that it is constitutively expressed in different mouse tissues but more abundantly in testis where it is also progressively up-regulated during the first spermatogenesis [44].

Here we report that the mouse genome presents two *Tbca* genes, one localized in chromosome 13 and one in chromosome 16 (*Tbca*16 - previously unidentified). The study of the tissue-specific expression of the *Tbca*13 and *Tbca*16 genes showed them to be ubiquitously expressed. Furthermore, during spermatogenesis, *Tbca*13 and *Tbca*16 present opposite patterns of expression. We also demonstrate that *Tbca*16 codes for a sense transcript and a cis-NAT *in vivo*, during spermatogenesis, and these transcripts are involved in the regulation of *Tbca*13 expression in a mouse spermatocyte cell line.

## Results

### The mouse genome contains two *Tbca* genes localized in chromosomes 13 and 16

**Figure 1:**
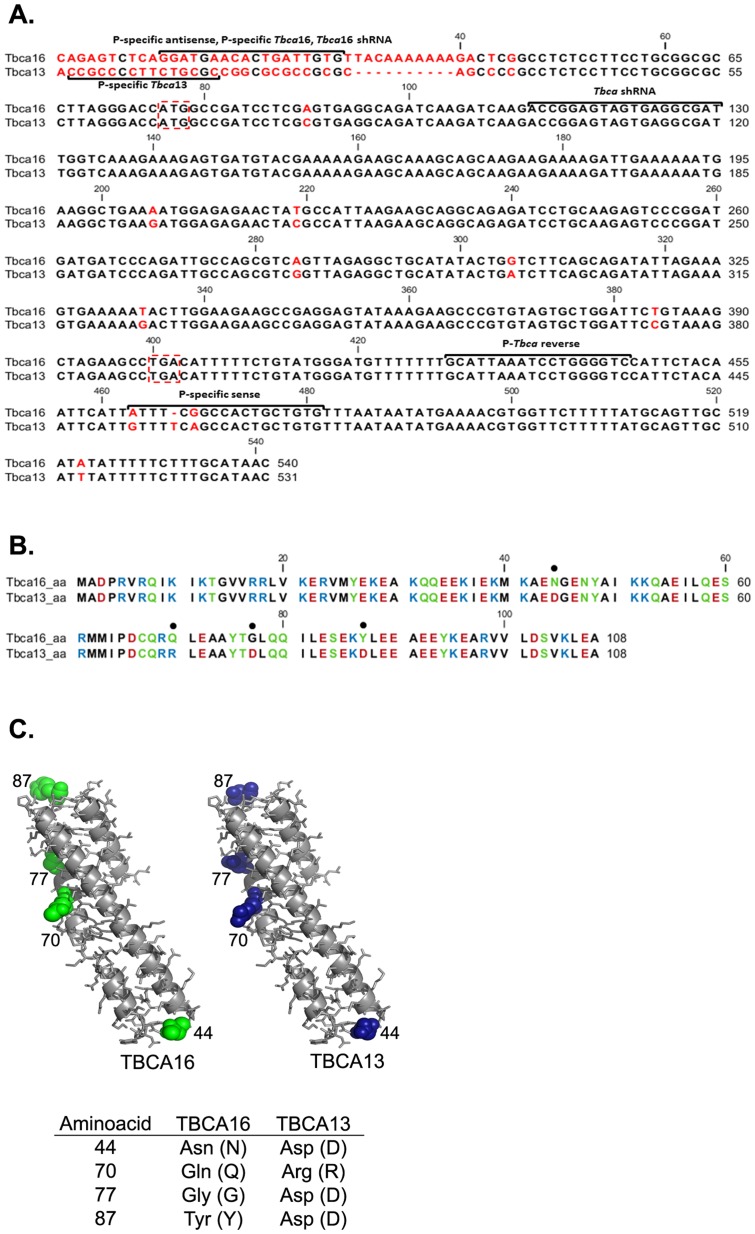
Comparison of *Tbca*13 and *Tbca*16 sequences and 3D Model Structure of TBCA16 and TBCA13. Comparison between nucleotide sequences of *Tbca*16 and *Tbca*13 (A). The alignment shows 7 differences inside the coding region (the different nucleotides are in red, start and stop codons are inside a red dashed box). The regions where the different primers/shRNAs were designed are indicated. (B) Comparison of the aminoacid sequences of the putative TBCA16 and theTBCA13 protein, aminoacids are colored according to their polarity. The 4 differences are signaled with a black dot above the respective aminoacid (B). The sequences in (A) and (B) were done using the CLC Sequence Viewer 6.5.3 Program. (C) TBCA13 and TBCA16 3D models obtained using the program PyMOL [55] program (C). The aminoacid differences are indicated in the table, accordingly to the aminoacid position.

TBCA is a β-tubulin binding protein that participates in the tubulin folding pathway. *In vi*tro, TBCA is not critical for tubulin folding but enhances β-tubulin dimerization [41]. On the other hand, our previous studies of TBCA loss-of-function showed that human TBCA is an essential gene in human cell lines, its depletion causing a decrease in α- and β-tubulin levels [43]. These data indicate that TBCA is important for microtubule formation and consequently for microtubule-dependent functions. This is also hinted by the fact that TBCA is highly expressed in testis and is up-regulated during spermatogenesis, a process in which the microtubule cytoskeleton is preponderant. These observations lead us to go further in the functional characterization of *Tbca in vivo*. In the course of these studies we have detected that the mouse genome possess two *Tbca* genes, one previously described localized in chromosome 13 (*Tbca*13) and an uncharacterized *Tbca* gene in chromosome 16 (*Tbca*16). *Tbca*16 is localized inside the intron 3–4 of the Adenylatecyclase 9 gene (*Adcy*9) being its putative coding sequence in the same strand as the *Adcy*9 exons. The nucleotide sequence analysis of the two *Tbca* genes revealed that their structure is different. The *Tbca13* gene presents its coding region interrupted by three introns. In contrast the *Tbca*16 gene is intronless. A comparison between the nucleotide sequences of both genes revealed that their coding sequences present a high degree of identity (98%) with only 7 nucleotide substitutions (Fig. 1A). This high degree of nucleotide sequence identity extends to the 5′and 3′-noncoding regions. For example, upstream of the ATG codon, the two genes present indistinguishable nucleotide sequences over 30 nucleotides and present an identity of 97% over 174 nucleotides of the 3′-non-coding region (Fig. 1A). As a consequence, the two *Tbca* genes encode two putative closely related proteins differing only in 4 aminoacid residues (Fig. 1B). These substitutions may not have significant impact in the 3D structure of the putative TBCA16 protein, given the similarity of their predicted 3D structures (Fig. 1C). Even so, important charged residues for putative protein interactions would disappear in the hypothetical TBCA16 protein.

### Tbca13 and Tbca16 present distinct patterns of expression during mouse testis maturation

**Figure 2:**
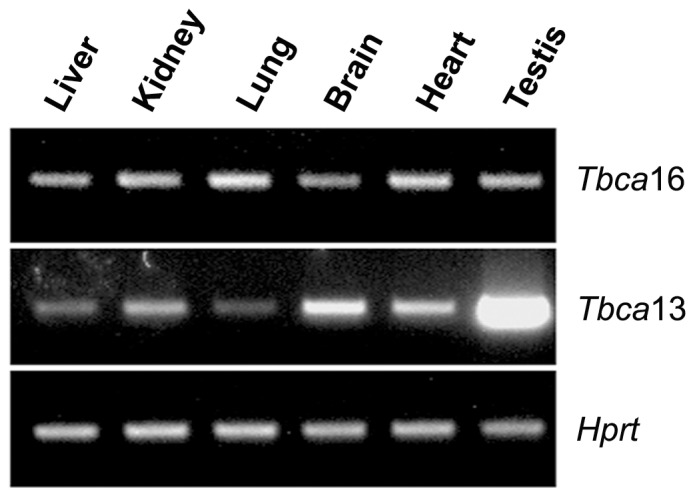
Study of *Tbca*16 and *Tbca*13expression in different mouse tissues by RT-PCR. RT-PCR analysis of *Tbca*13 and *Tbca*16 transcript levels using total RNA samples extracted from different mouse tissues (3 mice, 25 post-natal days old, were used in this analysis). In contrast with *Tbca*13 expression, *Tbca*16 is not highly expressed in testis. The *Hprt* expression was analyzed as an endogenous control.

**Figure 3:**
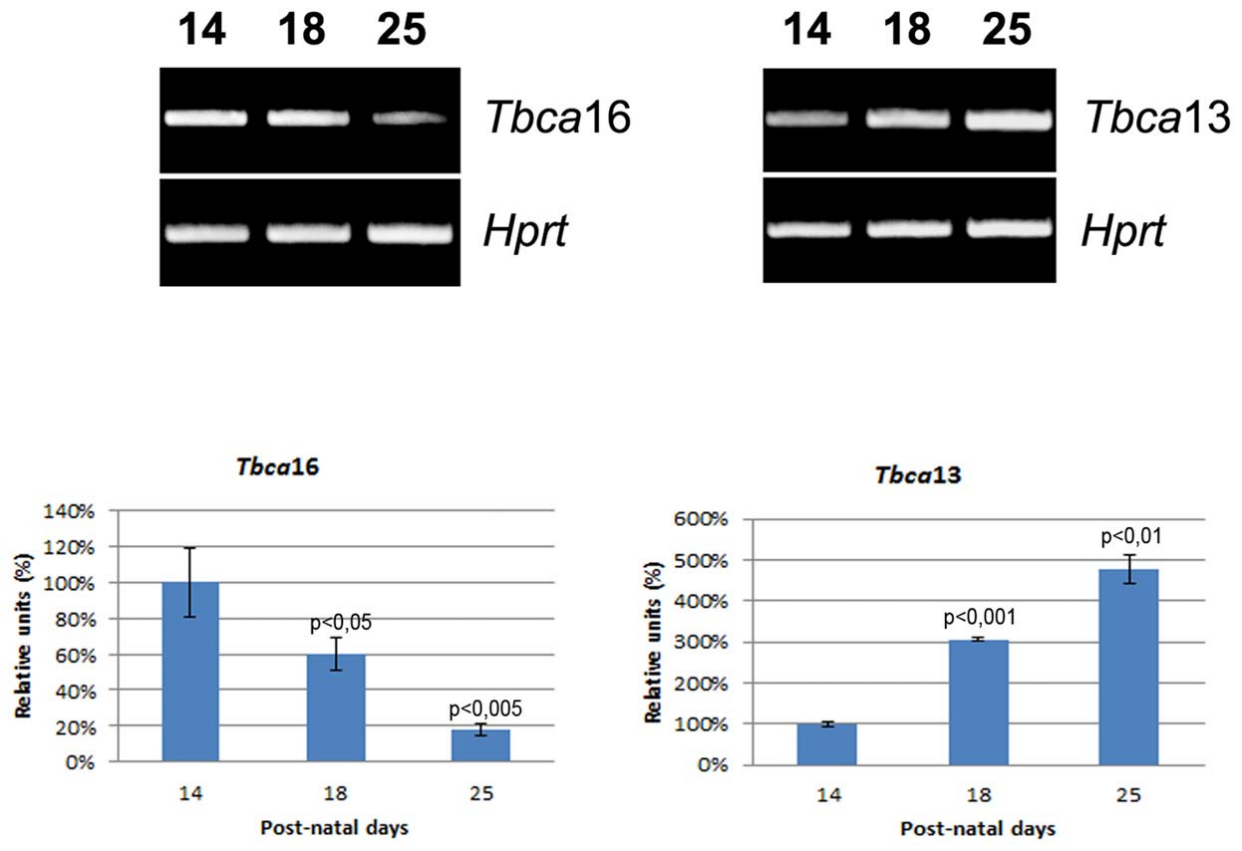
Study of *Tbca*16 and *Tbca*13 expression pattern during mouse spermatogenesis. Total RNA was obtained from mouse testis at different stages of maturation (3 sets of 14, 18 and 25 post-natal days old mice were used in this study). The *Tbca*16 and *Tbca*13 expression levels were analyzed by semi-quantitative RT-PCR and normalized by the expression of *Hprt*. During spermatogenesis the steady-state levels of *Tbca*13 mRNA increase whereas a decrease in the steady-state levels of *Tbca*16 mRNA is observed. Normalized cDNA levels are expressed as a percentage of maximal value (100%) for the 14 post-natal day. The p values were determined in comparison to the 14 post-natal days. The graphic bars are the mean±s.d. (error bars) of three independent assays. Statistical significance was calculated using a t-test.

Previous studies, where no distinction between the two specific *Tbca* transcripts was made, have shown that *Tbca* was more abundantly expressed in testis than other adult tissues. In fact, *Tbca* was progressively up-regulated from the onset of meiosis throughout spermatogenesis, being more abundant in differentiating spermatids [44]. This *Tbca* expression pattern was proposed to be associated to microtubule cytoskeleton changes and β-tubulin processing throughout spermatogenesis rather than to meiosis [44]. The existence of two mouse genes encoding two distinct TBCA isotypes prompted us to investigate if both genes were transcribed and presented tissue-specific regulation. Therefore, we extracted total RNA from different mouse organs and performed RT-PCR using specific forward primers for each *Tbca* gene. Each specific primer was designed to hybridize with a sequence located about 66 bp upstream of the start codon at 5′-UTR (5′-untranslated region see Fig. 1A, P-specific *Tbca*16) of the two *Tbca* genes, where these sequences started to diverge. Before any analysis, a search of each of these specific primers throughout the mice genome sequence was performed. This BLAST analysis showed these primers to be specific for the 5′-UTR of each *Tbca* gene. The reverse primer was the same for both genes and hybridizes with a conserved sequence localized close to the stop codon (see Fig. 1A. P-*Tbca* reverse). The obtained amplification products were sequenced and this analysis showed that these products correspond to the amplification of the expected transcripts from the gene under analysis. To exclude the possibility of genomic DNA contamination affecting these results, all RNA samples were treated with DNase I and tested by PCR using primers specific for genomic DNA, prior to cDNA synthesis. The obtained results showed that *Tbca*13, as well as *Tbca*16 are both transcribed at different levels in all the mouse organs analyzed (Fig. 2). This analysis also revealed that, contrary to *Tbca*16, *Tbca*13 is highly expressed in adult testis in comparison with the other organs. This shows that the high TBCA levels previously observed in mature testis [44] correspond to *Tbca*13. Given what was previously described we decided to analyze also the expression of *Tbca*13 and *Tbca*16 genes during the different stages of the mouse first spermatogenesis, which occurs during the first post-natal month in parallel with testis maturation. Consequently, we extracted total RNAs from mouse testis at different post-natal days (14, 18 and 25 days) and analyzed the expression of both *Tbca* genes by RT-PCR using the primers already described (Fig. 3). With this analysis we observed that *Tbca*13 and *Tbca*16 genes present opposite expression patterns during the process of testis maturation. In fact, *Tbca*13 mRNA levels increase progressively, while *Tbca*16 mRNA levels decrease. Taken together, these data indicate that the two genes are regulated differentially and suggest that the products encoded by them might play distinct roles during testis development.

### *Tbca16* is transcribed as a sense and an antisense RNA

**Figure 4:**
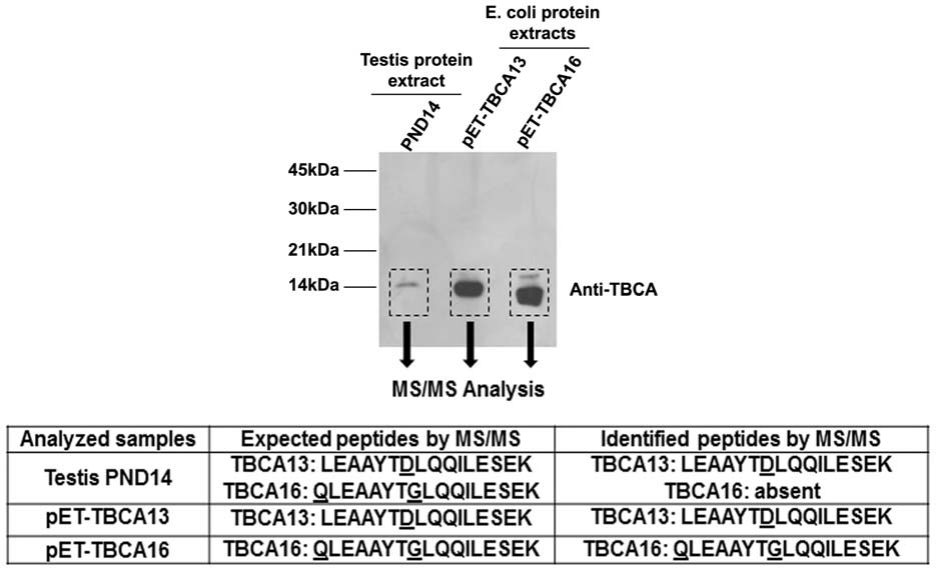
TBCA16 protein is absent in early stages of mouse spermatogenesis. Protein extracts from mouse testis in early stages of spermatogenesis (14 post-natal days – PND14) and recombinant TBCA13 and TBCA16 proteins produced and purified from bacteria were analyzed by 16.5% (w/v) Tricine–SDS–PAGE followed by western blot with a specific polyclonal antibody directed to TBCA. Note that this antibody recognizes both recombinant proteins. Under these conditions, TBCA13 and TBCA16 proteins have distinct motilities. The approximate molecular mass of the proteins is indicated at the left side of the panels. The regions around the 14 kDa marked by dashed squares were excised and proteins present in these regions were analyzed by electrospray mass spectrometry (MS/MS). The analyses lead to the identification of the TBCA13 specific peptide R.LEAAYTDLQQILESEK.D (the specific aminoacid is underlined). No TBCA16 specific peptides were identified (see table).

The fact that both *Tbca*13 and *Tbca*16 genes present distinct expression patterns during testis development led us to investigate if the steady-state levels of the respective encoded proteins accompanied the levels of the specific transcripts. In fact, the production of an mRNA encoded by the *Tbca*16 gene did not imply by itself the existence of a TBCA16 protein. Given the fact that the TBCA13 and TBCA16 predicted proteins differ only in 4 amino-acid residues it would be difficult to produce a specific antibody capable of distinguishing them. To overcome this difficulty and to identify the TBCA16 protein *in vivo* we cloned the coding regions of *Tbca*13 and *Tbca*16 in a *bacterial* expression vector and expressed them in *E. coli*. The recombinant proteins were purified and analyzed by electrophoresis on a Tricine-SDS-PAGE in parallel with a 14 post-natal days testis protein extract. This testis developmental stage was chosen since it corresponds to the stage with the highest *Tbca*16 mRNA levels (Fig. 3). This analysis was followed by western blot using an antibody against human TBCA that recognized the mouse TBCA13 and TBCA16 proteins produced in bacteria (Fig. 4). Interestingly, although both proteins present the same predicted molecular mass, under the conditions used, TBCA16 migrated faster than TBCA13 (Fig. 4). Thus, it was possible to distinguish the two proteins due to their distinct mobility in Tricine-SDS-PAGE. However, in 14 post-natal days testis protein extracts the antibody only recognized a unique protein band presenting a migration behavior similar to that of TBCA13 (Fig. 4). This suggested that, although these protein extracts corresponded to the testis maturation stage were *Tbca*16 is up-regulated, they only appeared to contain the protein product of the *Tbca*13 gene. To confirm this result we first re-analyzed protein extracts from 14 days old testis in parallel with purified TBCA13 and TBCA16 proteins in a Tricine-SDS-PAGE followed by Coomassie blue staining. In this analysis the purified TBCA13 and 16 proteins were used as migration references to allow the identification of the region where both TBCA proteins should be present in the analyzed testis protein extracts. Subsequently, this region was excised from the gel and the proteins presented there were identified by tandem mass spectrometry. To make sure that this analysis was able to distinguish between the two TBCA proteins, purified TBCA13 and 16 proteins were previously analyzed. The obtained data revealed that the distinction between TBCA13 and TBCA16 proteins by tandem mass spectrometry was possible due to the aminoacid substitutions occurring in position 77 and 88. In fact, the theoretical complete trypsin hydrolysis of the two TBCA proteins originated two distinct peptides with different molecular masses (TBCA13:LEAAYT**D**LQQILESEK and TBCA16:**Q**LEAAYT**G**LQQILESEK). Since the identification of the proteins analyzed by tandem mass spectrometry requires a search in protein databases to identify the specific protein profile we have updated them by introducing the corresponding TBCA16 data. Noteworthy, with this approach we were only able to detect the specific peptide corresponding to the TBCA13 protein in protein extracts of 14 days post-natal mouse testis (Fig. 4). The LEAAYT**D**LQQILESEK peptide was identified with a peptide score of 80, clearly above the identity threshold (51) determined by the search algorithm, thus confirming the unambiguous identification of TBCA13 in the sample. Although the peptide **Q**LEAAYT**G**LQQILESEK was not detected, the possibility of minimum amounts of TBCA16 being synthesized cannot be completely excluded. Furthermore this result is in agreement with the ones obtained by western blot (Fig. 4) indicating that the TBCA16 protein is most probably not being synthesized in this testis maturation stage.

**Figure 5:**
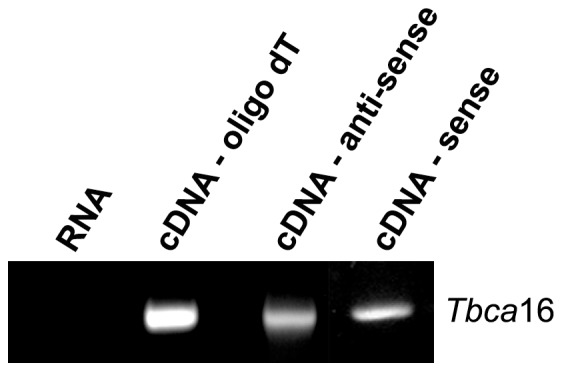
*Tbca*16 gene codes for a sense and for an antisense transcript. Total RNA was extracted from 14 post-natal days mouse testis and analyzed by RT-PCR. For the cDNA synthesis oligodT (**cDNA-oligodT**), *Tbca*16 sequence-specific primer for antisense orientation (**cDNA-antisense**) or *Tbca*16 sequence-specific primer for sense orientation (**cDNA-sense**) were used. The presence of the *Tbca*16 transcript was analyzed by PCR using specific primers. In every case a single band with the expected size was detected. A PCR performed only with RNA (**RNA**) as the template was done as a control to detect any residual amplification from a DNA genomic contamination.

These intriguing results associated with the observation that *Tbca*16 presents an opposite expression pattern to that of *Tbca*13 lead us to put forward the hypothesis that *Tbca*16 transcripts could play a regulatory role in the expression of the *Tbca*13 gene. Moreover, there are growing evidences in the literature showing that the expression of genes can be regulated by non-coding antisense mRNAs, some transcribed from their pseudogenes [29]. In our previous strategy to analyze the specific expression of *Tbca*13 and *Tbca*16 genes by RT-PCR (Fig. 2 and 3) the cDNAs were synthesized using a primer d(T) to hybridize with the poly(A)^+^ tail, which did not allow us to account for the orientation of the RNAs under study. To address this issue we set up an RT-PCR experiment in which the cDNA would be produced using a sequence-specific primer complementary to the *Tbca*16 mRNA in a sense (P-specific sense) and antisense orientation (P-specific antisense) (see Fig. 1A). Subsequently, we used DNase-treated RNAs from 14 post-natal days mouse testis to synthesize cDNA either using the specific sense- and antisense-*Tbca16* primers, or the universal d(T) primer. Next, *Tbca*16 transcripts were amplified by PCR using the specific primers for *Tbca*16 (Fig. 1A, P-specific *Tbca*16) and using the following templates: (i) DNase treated RNA used in the cDNA synthesis; (ii) a cDNA synthesized using the d(T) primer and (iii) cDNAs synthesized with the specific *Tbca*16 primer in sense or antisense orientation. The results presented in Fig. 5 show that there is no amplification when the RNA sample is used as a template showing that the RNA sample was not contaminated with genomic DNA. On the other hand, for each cDNA sample used, a single band with the expected size for the *Tbca*16 transcript was detected (Fig. 5). These PCR products were sequenced and their nucleotide sequence was an exact match to the sequence of *Tbca*16 transcripts leading to the conclusion that the *Tbca*16 gene is transcribed both in a sense and an anti-sense (anti-*Tbca*16) orientation.

The existence of two distinctly oriented transcripts from *Tbca*16 gene is noteworthy. In fact due to their perfect complementary sequence it is conceivable that they can be paired *in vivo*, originating long double strand RNA molecules. These molecules have been described to be precursors for the interference RNA machinery leading to the formation of siRNA duplexes [34], [35]. In this context we put forward the hypothesis that the *Tbca16* gene is a regulator of the *Tbca13* causing it's silencing by originating a population of sequence-specific siRNAs for *Tbca*13 mRNAs.

### The steady-state levels of the *Tbca*16 transcripts affect the levels of *Tbca*13 mRNA

To investigate the putative regulatory role of the sense and anti-sense *Tbca16* in the levels of the *Tbca13* transcripts *in vivo*, we decided to perform RNAi experiments. For this purpose we designed a shRNA targeting specifically the sense and anti-sense *Tbca*16 transcripts (see fig. 1A, *Tbca*16 shRNA). In this experiment we also designed a shRNA targeting both *Tbca* genes (*Tbca* shRNA). Unfortunately, specific shRNAs targeting the *Tbca*13 transcripts were not effective in the knockdown process.

**Figure 6:**
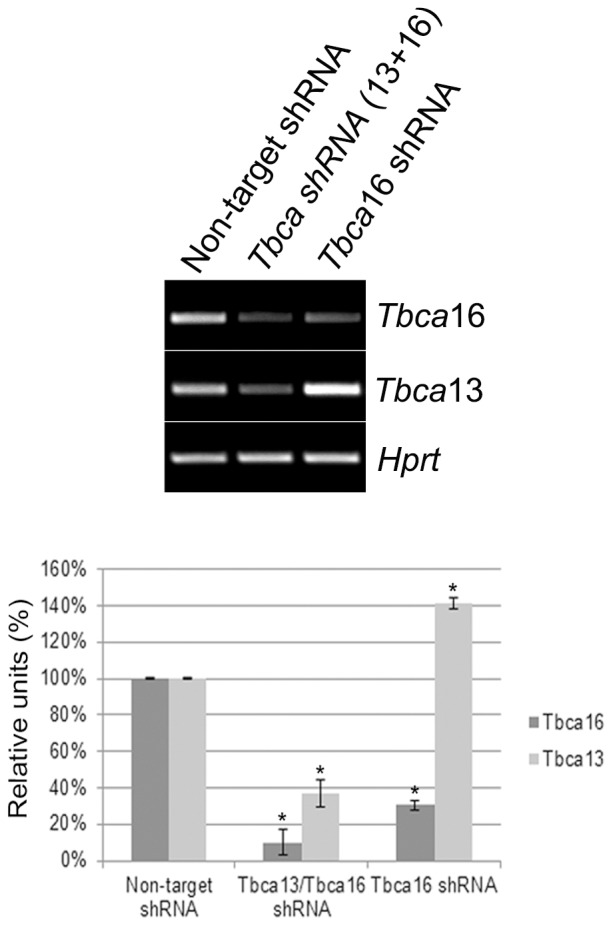
*Tbca*16 knockdown increases the steady-state levels of *Tbca*13 RNA in GC-2spd(ts)-spermatocyte mouse cell line. After 48 h of transfection, total RNA was extracted from GC-2spd(ts) cells expressing non-target shRNA, *Tbca*shRNA(knockdown*Tbca*13+*Tbca*16) or *Tbca*16shRNA (to knockdown exclusively the *Tbca*16 RNA). Semi-quantitative RT-PCR analysis showed a decrease in the steady-state levels of *Tbca*13 and *Tbca*16 mRNAs in *Tbca* shRNA expressing cells in comparison to those in cells expressing non-target shRNA. However, in cells expressing *Tbca*16 shRNA the steady-state levels of *Tbca*16 mRNA decrease whereas those of *Tbca*13 mRNA increase. Normalized cDNA is expressed as a percentage of maximal value (100%). *p<0,05 compared with control. The graphic bars are the mean±s.d. (error bars) of three independent assays. Values were standardized with those of *Hprt* cDNA. Statistical significance was calculated using a t-test.

Taking into consideration the expression patterns of the two *Tbca* genes during testis maturation we decided to study the effects of the specific *Tbca*16 knockdown in the GC-2spd(ts)-spermatocyte mouse cell line (GC-2 cells). As a control, GC-2 cells were transfected with a plasmid encoding a non-target shRNA to check for general non-specific effects associated with shRNA delivery and to confirm the sequence specificity of the silencing effect (negative control). Additionally, GC-2 cells were transfected with the *Tbca* shRNA coding plasmid to target the transcripts of both *Tbca* genes (13+16) and with a plasmid coding for the *Tbca*16 shRNA specifically targeting the transcripts of the *Tbca*16 gene. At 48 h post-transfection total RNAs were extracted from GC-2 cells expressing the non-target shRNA, the *Tbca*(13+16) shRNA, or the *Tbca16*shRNA which were then analyzed by RT-PCR. Semi-quantitative RT-PCR analysis showed a decrease in the steady-state levels of *Tbca16* mRNA in *Tbca*(13+16) and in *Tbca16*shRNA expressing cells, in comparison with those found in control cells expressing the non-target shRNA (Fig. 6). Also, a decrease in the *Tbca*13 mRNA steady-state levels was observed in cells transfected with the *Tbca*(13+16) shRNA. Remarkably, in cells expressing the *Tbca*16 shRNA we observed an increase in *Tbca*13 mRNA steady-state levels to levels higher than those found in cells expressing the non-target shRNA. The difference observed in *Tbca*16 mRNA decrease levels in cells transfected with *Tbca*(13+16) shRNA and *Tbca*16 shRNA could be explained by different silencing efficiencies of both interfering RNAs. Also, when GC-2 cells are transfected with a recombinant plasmid (see Material and Methods) to over-express the anti*Tbca*16 transcript we observed an increase in the anti*Tbca*16 transcript levels. The observed increase is accompanied by a decrease of about 20% on the *Tbca*13 transcript levels (Fig. S1).

To investigate if the alterations in the transcript levels caused by RNAi depletion of *Tbca*16 transcripts have an impact in TBCA protein levels we have also performed a western blot analysis of soluble protein extracts isolated from GC-2 cells transfected either with *Tbca*(13+16) shRNA or *Tbca*16 shRNA for 48 h (Fig. S2). The results showed a slight decrease in the steady state levels of the TBCA protein when GC-2 cells are transfected with the *Tbca*(13+16) shRNA, whereas an increase of TBCA protein levels was detected when cells were transfected with *Tbca*16 shRNA for the same period of time (Fig. S2) which shows again the regulatory role of the *Tbca*16 RNAs.

All together the results strongly support our hypothesis that the *Tbca*16 transcripts are involved in the post-transcriptional regulation of the *Tbca*13 gene expression. Moreover, these results can explain the opposite expression patterns of the two *Tbca* genes during spermatogenesis.

## Discussion

Some pseudogenes are transcriptionally active producing noncoding RNAs that are able to regulate the expression of functional genes by a variety of mechanisms [29]. NATs are noncoding regulatory RNAs that occur ubiquitously in prokaryotes and eukaryotes carrying out critical functions. The analysis of the validated NAT lists showed that many are transcribed from genes involved in development or associated with human diseases [27].

In this work we identified a mouse intronless *Tbca* gene localized in chromosome 16 (*Tbca*16) that is transcribed in both orientations originating sense and anti-sense transcripts. This gene is closely related to the mouse *Tbca*13 localized in chromosome 13 that has three introns and encodes TBCA, a protein involved in tubulin heterodimer maturation. In fact, both genes share 97% of nucleotide sequence identity in their coding regions and are highly identical throughout part of their 5′and 3′non-coding regions. Since the *Tbca*16 gene is transcribed in the sense orientation it may encode a TBCA16 protein differing from the TBCA13 protein in only 4 amino-acid residues. Interestingly, we observed that the steady-state levels of *Tbca*16 transcripts (sense and anti-sense) decrease during testis maturation while the *Tbca*13 transcript levels increase. These observations led us to investigate the presence of the TBCA16 protein in protein testis extracts corresponding to the developmental stages where *Tbca*16 was up-regulated during testis maturation. However, all attempts to identify the putative TBCA16 protein, either by western blot or mass spectrometry, have failed suggesting that most probably the TBCA16 protein is not translated or is very unstable. These unexpected observations led us to reassess the role of the *Tbca*16 gene.

Taking into account the analysis of the structure and nucleotide sequence features of the *Tbca*16 gene, in comparison with those of the *Tbca13* gene, it is plausible that *Tbca*16 gene could have been generated by a retrotransposition event of processed *Tbca13* transcripts that in this case would be the parental gene. Indeed, according to a study focused in the origin of human retrotransposed mRNAs [45] the *Tbca*13 mRNA characteristics fit extremely well in the class of mRNAs that are prone to create processed pseudogenes. Moreover, it is also generally accepted that germline expression is crucial for the inheritance of processed pseudogenes and the *Tbca13* gene is highly expressed in testis. Moreover, if this hypothesis is true than the event/s that led to the creation of the mouse *Tbca16* pseudogene most probably occurred before the divergence at least of the mammals, because *Tbca*16 homologous intronless genes were found in the rat (chromosome 7), human (chromosome X) and chimpanzee (chromosome X) genomes. Also, a close analysis of the human *Tbca*X nucleotide sequence shows that the coding region of this gene is interrupted by a stop codon (TAA) in position 64, supporting the idea that this gene if transcribed and translated produces a truncated, probably non-functional protein and therefore is probably a pseudogene. The conserved presence of pseudogenes in the genome of related species has been used as an argument to support the idea that conserved pseudogenes have been maintained due to having a functional role [29].

Furthermore, the opposite pattern of expression of *Tbca*16 and *Tbca*13 during testis maturation suggested that there is a regulatory mechanism between *Tbca*16 and *Tbca*13 transcript levels. This hypothesis is strongly supported by the fact that the specific decrease of *Tbca*16 transcripts by RNAi in the spermatocyte mouse cell line GC-2 lead to an increase in *Tbca*13 transcripts. In accordance the overexpression of these transcripts causes a decrease in *Tbca*13 transcripts (Fig. S1). Consequently, *Tbca*16 transcripts may play a critical role in the post-transcriptional regulation of *Tbca*13 gene expression. In fact, the depletion of Tbca16 transcripts from GC-2 cells by RNAi causes an increase in TBCA protein levels (Fig. S2) Although, the exact mechanism behind this regulation remains to be elucidated, the fact that the two *Tbca* genes share a high degree of nucleotide sequence identity suggest that this may occur by a mechanism of RNA interference [34], [35]. In this view the antisense-*Tbca*16 RNA would pair with the sense-*Tbca*16 transcript and also with *Tbca*13 mRNA forming long double-stranded RNA molecules *in vivo*, that then would be processed by the endogenous RNAi machinery originating specific siRNAs that would interfere with *Tbca*13 transcripts. In agreement with this idea, recent studies in plants and mouse identified endo-siRNAs involved in the regulation of gene expression and that were derived from sense-antisense RNA pairs (pseudogene-pseudogene and pseudogene with 90% homology-coding mRNA) produced through the RNA interference pathway [34], [35], [46].

It has been proposed, based on large scale genomic approaches, that NATs overexpression in testis is a general phenomenon and not restricted to specific genes [47], [48]. Moreover, most of the transcribed pseudogenes are expressed in testis [37]. The biological significance of these events in testis is far from being understood, however it is tempting to propose that these regulatory mechanisms are an additional guarantee for the correct development and specific function of this highly differentiated organ that originates the germ-line. Accordingly, the *Tbca*13 and *Tbca*16 genes opposite expression pattern during testis maturation, associated to the fact that *Tbca*13 mRNA protein levels, are affected by the amount of the *Tbca*16 transcripts in a spermatocyte mouse cell line, strongly support the idea that the specific regulation between the two types of transcripts is important for organ function/maturation. Spermatogenesis is a very complex developmental process that requires precise microtubule cytoskeleton remodeling originating complex microtubule structures like the manchette and the flagellum of the sperm [38]. Therefore, it is conceivable that TBCA plays an important role during this process and that the fine-tuned regulation of TBCA levels is critical and achieved by a post-transcriptional regulation mechanism involving the expression of *Tbca*16 transcripts.

## Materials and Methods

### Ethics statement

In total, 12 Swiss-Webster mice were used in this study. To extract RNA from testis of different ages, 3 sets of 3 mice, each set with one mouse of 14, 18 and 25 post-natal days old were used (figure 3). From the adult mice (25 post-natal days) we also extracted RNA from different organs (figure 2). Then, 3 additional mice with 14 post-natal days old were used to obtain protein extracts from testis for the mass spectrometry analysis. They were killed according to PHS policy and the U.S. National Institutes of Health guidelines. The animal experiments were approved by the ethical committee from “Universidad da Cantabria”, Spain. The approval documents are signed by the president of the ethical committee, Professor Miguel Lagarfa Coscojuela, and were uploaded in the “attach files section” together with the manuscript (as a JPEG files).

### Cell culture

GC-2spd(ts) cell line (mouse spermatocyte; SV40 large T antigen transfected; these cells are arrested at a premeiotic stage – this cell line was purchased from ATCC-LGC [49]) was cultured in a 5% CO_2_ humidified atmosphere at 37°C as exponentially growing sub-confluent monolayers in Dulbecco's modified Eagle's medium (DMEM) with Glutamax (Invitrogen), supplemented with 10% fetal calf serum (Invitrogen).

### RNA extraction and RT-PCR expression analysis

Total RNA was extracted from mice organs and also from GC-2 cell line using the RNeasy mini-kit (Qiagen), according to the manufacturer's protocol. The RNA was treated with DNase I (Invitrogen) and reverse transcribed using SuperScript II (Invitrogen) and an oligo(dT)12–18 primer (Invitrogen) or using specific primers for *Tbca*16: sense (5′-CACAGCAGTGGCCGAAAT-3′) and antisense orientation (5′-GGATGAACACTGATTGTG-3′).

The RT-PCR procedure was adapted from [50]. The amount of cDNA in each sample was first normalized, after non-saturing PCR, for *Hprt* (hypoxanthine guanine phosphoribosyltransferase 1-standard internal control) transcripts. The identity of PCR products was confirmed by sequencing. Semi-quantitative RT-PCR expression analysis was performed using the following forward and reverse primers: mouse *Hprt* (Accession number – NM_013556) - 5′-GGACAGGACTGAAAGACTTG-3′ and 5′-CACAAACGTGATTACAATCCC-3′; mouse *Tbca*13 (Accession number – NM_009321) - 5′-CACCGCCCCTTCTGCGC-3′ and 5′-GACCCCAGGATTTAATGC-3′; mouse *Tbca*16 - 5′-GGATGAACACTGATTGTG-3′ and 5′-GACCCCAGGATTTAATGC-3′. To amplify *Tbca*13 and *Tbca*16, from different adult mice tissues (25 post-natal days), we have performed 36 cycles in the PCR amplification process in order to guarantee a detectable amplification product even in organs where *Tbca*13 and *Tbca*16 was less/vestigial expressed. Using these PCR conditions the expression of *Tbca*13 in testis corresponds to a saturated band that impairs any quantification. To avoid any saturation and allowing quantification the PCR reaction conditions were distinct in the experiments concerning the study of the expression of *Tbca*13 and *Tbca*16 during testis development. Thus we have performed 28 cycles for *Hprt* (internal control), 30 cycles for *Tbca*13 and 36 cycles for *Tbca*16 in the PCR amplification process.

### Protein extracts, TBCA purification and mass spectrometric analysis

cDNAs from *Tbca*13 and *Tbca*16 were cloned into the bacteria expressing vectors pET3a (Novagen). Recombinant TBCA13 and TBCA16 proteins were produced and purified from Escherichia coli BL21:DE3 cultures as previously described [51]. The recombinant proteins were used to optimize the tandem electrospray mass spectrometry conditions.

Mouse testis protein extracts (from 14 post-natal days) and GC-2 transfected cells with non-target siRNA, with *Tbca*(13+16) shRNA or *Tbca*16 shRNA during 48 h were performed accordingly to Nolasco et al., 2005 [43] and analyzed on a 16.5% (w/v) Tricine–SDS–PAGE [52] and the band correspondent to the TBCA expected region was excised manually from the gel and analyzed by tandem mass spectrometry. For peptide identification MASCOT (Matrix Science) search engine was used and peptide score threshold indicating identity or extensive homology was 51 (p<0.05 for an observed match to be a random event).

### Western blot analysis

Protein extracts were separated on a 16.5% (w/v) Tricine–SDS–PAGE [52]. Westerns blots were performed using the rabbit polyclonal sera against TBCA (1∶5000) [53] and Anti-Actin (20–33) antibody produced in rabbit (1∶2000) (Sigma). Secondary antibody against rabbit (Jackson Immuno Research) was used at 1∶4000. The immunostaining was carried out using the ECL technique (GE Healthcare). The molecular mass markers used were purchased from GE Healthcare.

### Construction of RNAi vectors, cloning of anti*Tbca*16 and transfection

pSUPER, vector used for expression of short interfering RNAs (shRNAs), was purchased from OligoEngine (Seattle, WA, USA) and the constructs were done as described previously [54]. To construct the pSUPER_*Tbca*_shRNA, pSUPER_*Tbca*16_shRNA and pSUPER_Non Target_shRNA vectors pairs of 64-nt oligonucleotides (Sigma), forward and reverse, each containing a unique 19-nt sequence derived from within the target mRNA transcripts (see below), was annealed and ligated between the BglII/HindIII sites of the pSUPER. Within the 64-nt oligomers, the 19-nt target sequence appears in both sense and anti-sense orientation (bold), separated by a 9-nt spacer sequence (underlined).

- pSUPER_*Tbca*_shRNA oligonucleotides: 5′GATCTCC**GACCGGAGTAGTGAGGCGA** TTCAAGAGA **TCGCCTCACTACTCCGGTC**TTTTTGGAAA3′ and 5′AGCTTTTCCAAAAA**GACCGGAGTAGTGAGGCGA** TCTCTTGAA **TCGCCTCACTACTCCGGTC**GGA3′.

- pSUPER_*Tbca*16_shRNAoligonucleotides:

5′GATCTCC**AGGATGAACACTGATTGTG** TTCAAGAGA **CACAATCAGTGTTCATCC**TTTTTTGGAAA3′ and 5′AGCTTTTCCAAAAAA**GGATGAACACTGATTGTG** TCTCTTGAA **CACAATCAGTGTTCATCCT**GGA3′.

- pSUPER_NonTarget_shRNA oligonucleotides:

5′GATCTCC**GATCAAGACCGAACAATCC** TTCAAGAGA **GGATTGTTCGGTCTTGATC**TTTTTGGAAA3′ and

5′AGCTTTTCCAAAAA**GATCAAGACCGAACAATCC** TCTCTTGAA **GGATTGTTCGGTCTTGATC**GGA3′.

pcDNA3, vector used to overexpress the anti*Tbca*16 cDNA, was purchased from Invitrogen. To construct the recombinant pcDNA3 anti*Tbca*16 we amplified anti*Tbca*16 using the primers previously described at the Material and Methods in the “RNA extraction and RT-PCR expression analysis” section but containing at their 5′ ends sequences for restriction enzymes: *Hind*III (for the reverse primer) and *Bam*HI (for the forward primer). Using these enzymes anti*Tbca*16 was cloned under the promoter region of the vector which will upon transcription will produce anti*Tbca*16 transcripts.Transfections were performed with lipofectamine 2000 (Invitrogen) as specified by the manufacturer. At 18 h prior to transfection, 8×10^4^ cells were seeded per well for a 6-well plate.

### Statistical analysis

The experiments were performed at least three times and the results were expressed as means ± S.D. Differences between the data were tested for statistical significance by t-test. P values less than 0.05 were considered statistically significant.

## Supporting Information

Figure S1 **Anti** ***Tbca*** **16 overexpression decreases the steady-state levels of** ***Tbca*** **13 RNA in GC-2spd(ts)-spermatocyte mouse cell line.** After 48 h of transfection, total RNA was extracted from GC-2spd(ts) cells overexpressing pcDNA3 (control) and the recombinant vectors pcDNA3_Anti*Tbca*16. Semi-quantitative RT-PCR analysis showed a decrease in the steady-state levels of *Tbca*13 mRNA whereas the *Tbca*16 transcript levels increase in comparison to control cells. In the graphic normalized cDNA is expressed as a percentage of values found in control cells transfected with pcDNA3. Values were normalized with those of *Hprt* cDNA. Graphic bars show mean values of two independent experiments.
(TIF)

Figure S2 ***Tbca*** **16 knockdown by RNAi increases the steady-state levels of TBCA protein in GC-2spd(ts)-spermatocyte mouse cell line.** After 48 h of transfection soluble protein extracts were prepared from GC-2spd(ts) cells expressing non-target shRNA, *Tbca*shRNA (knockdown *Tbca*13 and *Tbca*16 RNAs) or *Tbca*16shRNA (to knockdown exclusively the *Tbca*16 RNA) and analysed on a 16.5% (w/v) Tricine–SDS–PAGE and probed with a polyclonal antibody against human TBCA or a monoclonal against actin. Western blot analysis showed a decrease in the steady-state levels of the TBCA protein in *Tbca* shRNA expressing cells in comparison to those in cells expressing non-target shRNA. However, in cells expressing *Tbca*16 shRNA, the steady-state levels of TBCA protein increases. Normalized protein levels are expressed as a percentage of the values found in cells expressing non-target shRNA (control cells). Values were normalized with those of actin protein. Graphic bars show mean values of two independent experiments.
(TIF)
