# Supplementary figures and images for: The Expression of Tubulin Cofactor A (TBCA) Is Regulated by a Noncoding Antisense Tbca RNA during Testis Maturation
Source: PLoS One. 2012 Aug 6;7(8):e42536. doi: 10.1371/journal.pone.0042536 (PMC3412815; doi:10.1371/journal.pone.0042536)

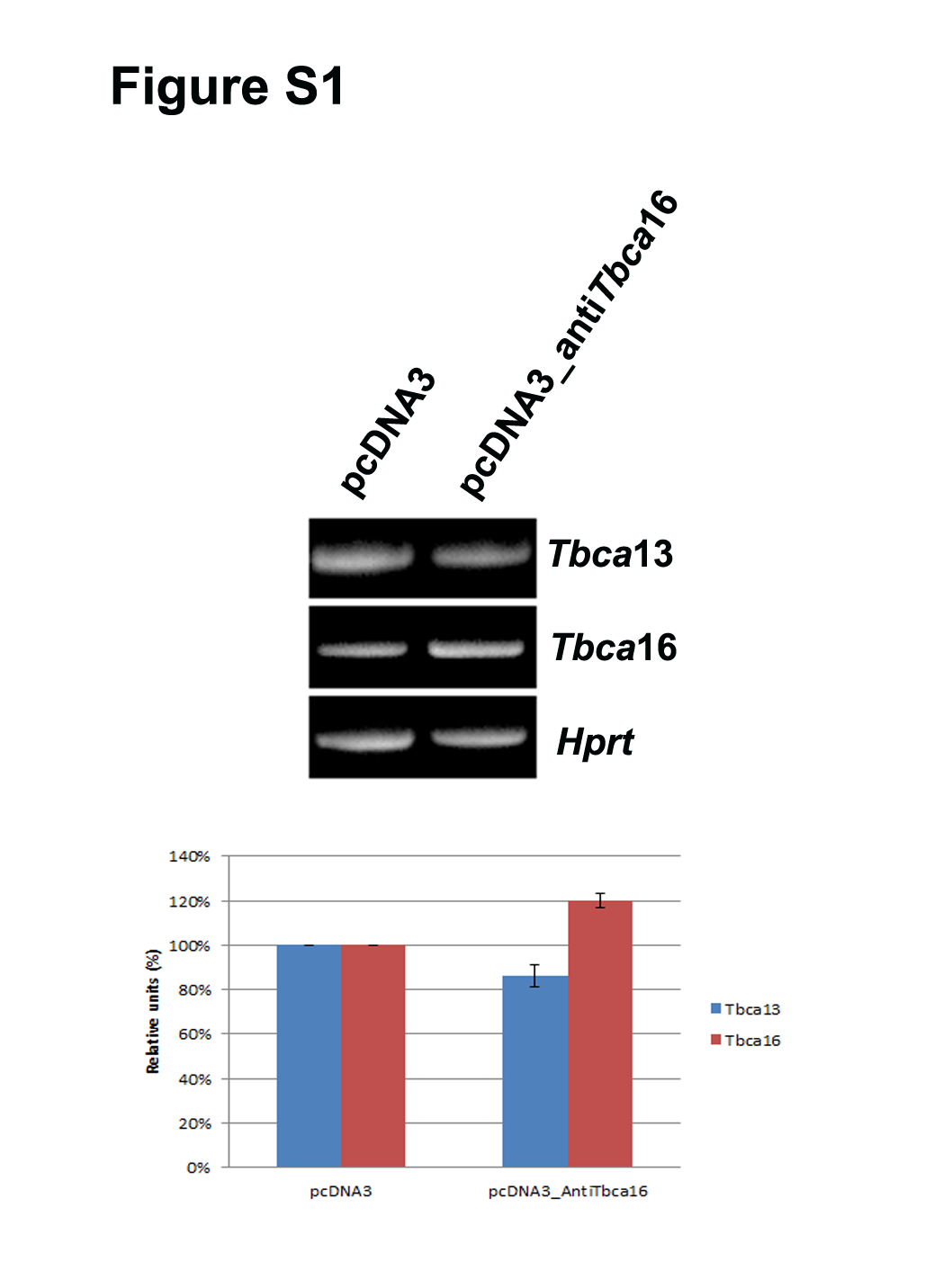

Supplement: Figure S1 — Anti Tbca 16 overexpression decreases the steady-state levels of Tbca 13 RNA in GC-2spd(ts)-spermatocyte mouse cell line. After 48 h of transfection, total RNA was extracted from GC-2spd(ts) cells overexpressing pcDNA3 (control) and the recombinant vectors pcDNA3_AntiTbca16. Semi-quantitative RT-PCR analysis showed a decrease in the steady-state levels of Tbca13 mRNA whereas the Tbca16 transcript levels increase in comparison to control cells. In the graphic normalized cDNA is expressed as a percentage of values found in control cells transfected with pcDNA3. Values were normalized with those of Hprt cDNA. Graphic bars show mean values of two independent experiments. (TIF) [file pone.0042536.s001.tif]

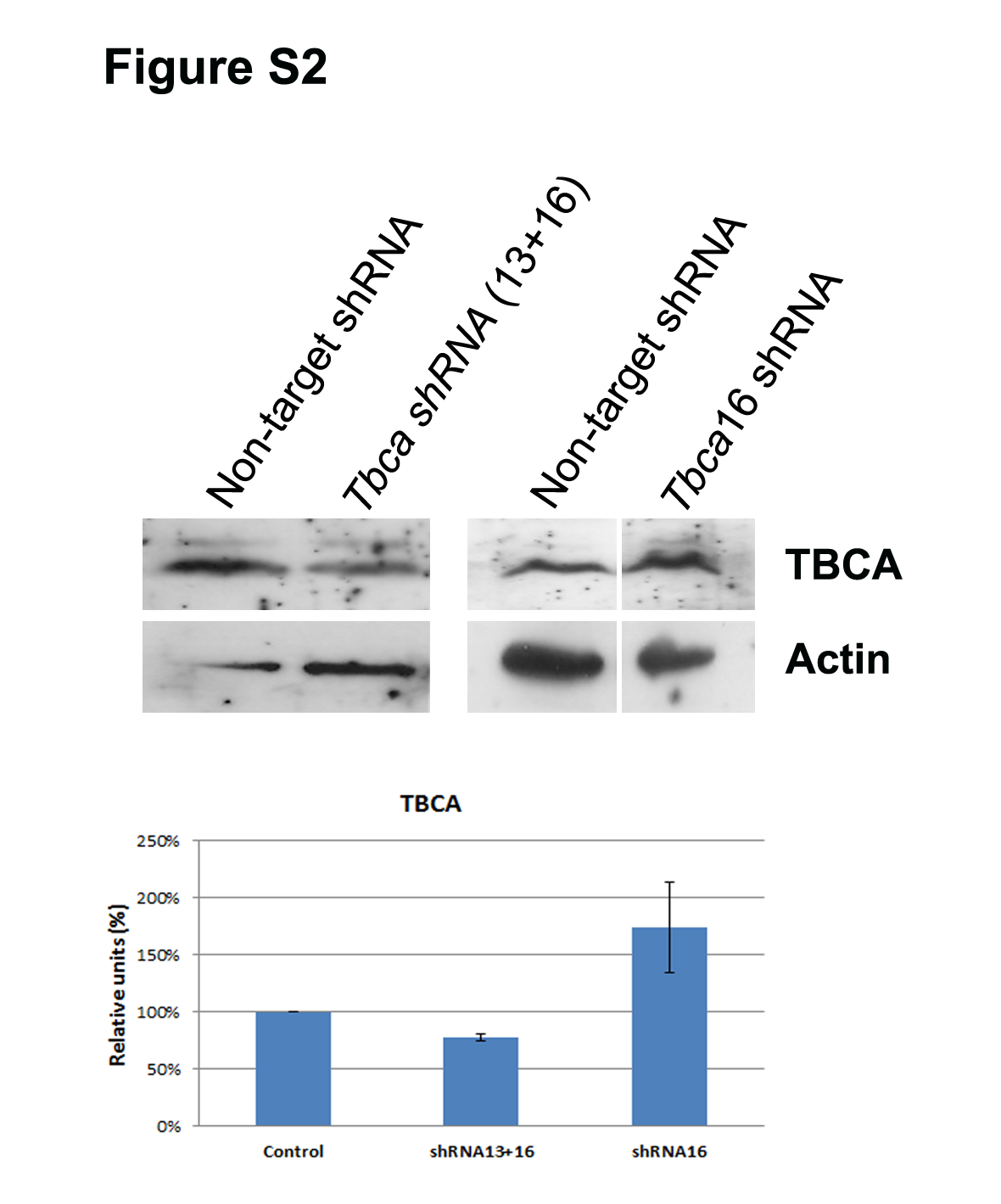

Supplement: Figure S2 — Tbca 16 knockdown by RNAi increases the steady-state levels of TBCA protein in GC-2spd(ts)-spermatocyte mouse cell line. After 48 h of transfection soluble protein extracts were prepared from GC-2spd(ts) cells expressing non-target shRNA, TbcashRNA (knockdown Tbca13 and Tbca16 RNAs) or Tbca16shRNA (to knockdown exclusively the Tbca16 RNA) and analysed on a 16.5% (w/v) Tricine–SDS–PAGE and probed with a polyclonal antibody against human TBCA or a monoclonal against actin. Western blot analysis showed a decrease in the steady-state levels of the TBCA protein in Tbca shRNA expressing cells in comparison to those in cells expressing non-target shRNA. However, in cells expressing Tbca16 shRNA, the steady-state levels of TBCA protein increases. Normalized protein levels are expressed as a percentage of the values found in cells expressing non-target shRNA (control cells). Values were normalized with those of actin protein. Graphic bars show mean values of two independent experiments. (TIF) [file pone.0042536.s002.tif]
